# Supplementary material for: Polydispersity vs. Monodispersity. How the Properties of Ni-Ag Core-Shell Nanoparticles Affect the Conductivity of Ink Coatings
Source: Materials (Basel). 2021 Apr 29;14(9):2304. doi: 10.3390/ma14092304 (PMC8125038; doi:10.3390/ma14092304)
Supplement: Supplementary file 1 [file materials-14-02304-s001.zip › materials-1182142-supplementary.pdf]

materials

MDPI

Supplementary Material

Polydispersity vs. Monodispersity. How the Properties of Ni-Ag Core-Shell Nanoparticles Affect the Conductivity of Ink Coatings

Anna Pajor-Świerzy [1,*], Dawid Staśko [1], Radosław Pawłowski [2], Grzegorz Mordarski [1], Alexander Kamyshny [3] and Krzysztof Szczepanowicz [1]

[1] Jerzy Haber Institute of Catalysis and Surface Chemistry Polish Academy of Sciences, Niezapominajek 8, 30239 Kraków, Poland; stasko.d@outlook.com (D.S.); nbmordar@cyf-kr.edu.pl (G.M.); ncszczep@cyf-kr.edu.pl (K.S.)

[2] Abraxas Jeremiasz Olgierd, Piaskowa 27, 44300 Wodzisław Śląski, Poland; radek.pawlowski@helioenergia.com

[3] Casali Center for Applied Chemistry, Institute of Chemistry, Edmond J. Safra Campus, The Hebrew University of Jerusalem, Jerusalem 91904, Israel; alexander.kamyshny@mail.huji.ac.il

* Correspondence: ncpajor@cyf-kr.edu.pl

Citation: Pajor-Świerzy, A.; Staśko, D.; Pawłowski, R.; Mordarski, G.; Kamyshny, A.; Szczepanowicz, K. Polydispersity vs. Monodispersity. How the Properties of Ni-Ag Core-Shell Nanoparticles Affect the Conductivity of Ink Coatings. *Materials* **2021**, *14*, 2304. https://doi.org/10.3390/ma14092304

Academic Editor: Arunas Ramanavicius

Received: 29 March 2021
Accepted: 26 April 2021
Published: 29 April 2021

Publisher's Note: MDPI stays neutral with regard to jurisdictional claims in published maps and institutional affiliations.

Copyright: © 2021 by the authors. Licensee MDPI, Basel, Switzerland. This article is an open access article distributed under the terms and conditions of the Creative Commons Attribution (CC BY) license (http://creativecommons.org/licenses/by/4.0/).

The effect of the complexing agents on the Ni NPs size distribution was investigated. As can be observed in Figure S1, in the presence of only citric acid, the slightly smaller (~220 nm) Ni NPs were obtained in comparison to the ones (~250 nm), which were obtained in synthesis without any complexing agent (Figure S1A). The greater decrease of the NPs diameter, from 250 to about 180 nm, was observed in the presence of both complexing agents (CA and AMP, Figure S1B, red curve). In addition, the smaller Ni NPs (of about 180 nm) were formed at a higher concentration of AMP, when pH was increased from 9.5 to 12.0. The bigger nanoparticles were obtained after the reduction process of Ni^{2+} in the presence of only one type of complexing agent (AMP or CA), which is presented in Figure S1C. The Ni NPs with the size of about 220 (green curve) and 400 nm (red curve) in the synthesis with CA and excess of AMP, respectively, were formed. The obtained results indicate, that the presence of both complexing agents affects the size of Ni NPs, which also suggests the formation of their mixed complex.

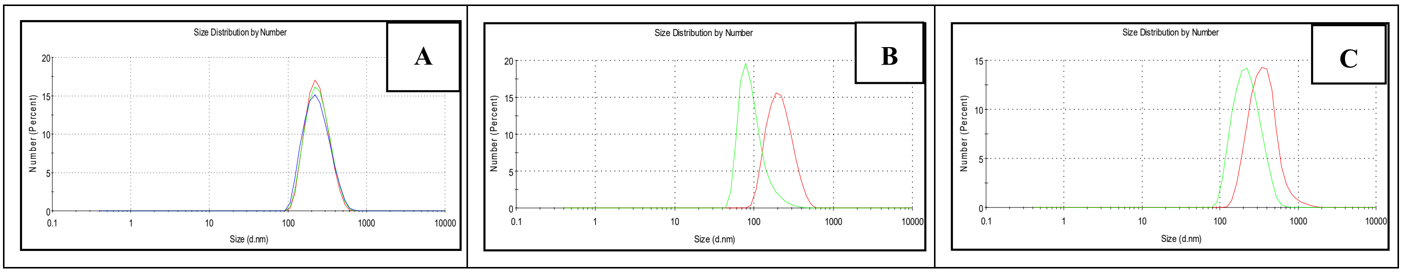

Figure S1. The size distribution of Ni NPs as the results of synthesis: (**A**) without complexing agents (Ni NPs ~250 nm); (**B**) with CA and AMP at pH ~9.5, Ni NPs ~80 nm (green curve) and pH ~12.0, Ni NPs ~180 nm (red curve); (**C**) with CA, Ni NPs ~220 nm (green curve) or excess of AMP, Ni NPs ~400 nm(red curve).

As can be seen in Figure S2, the most uniform coatings were obtained for inks containing 0.05% Ni-Ag NPs with an average size of 100 and 420 nm and for inks containing Ni-Ag NPs with an average size of 220 nm Ni-Ag NPs in the presence of TEGO WET KL 245 with concentration 0.025%. For inks containing the mixture of Ni-Ag NPs with different average sizes, the optimal concentration of wetting agent (for all mixing ratios) was found to be 0.035%. As was shown in Figure S2, the obtained metallic film is uniform, the holes or cracks were not visually recognized.

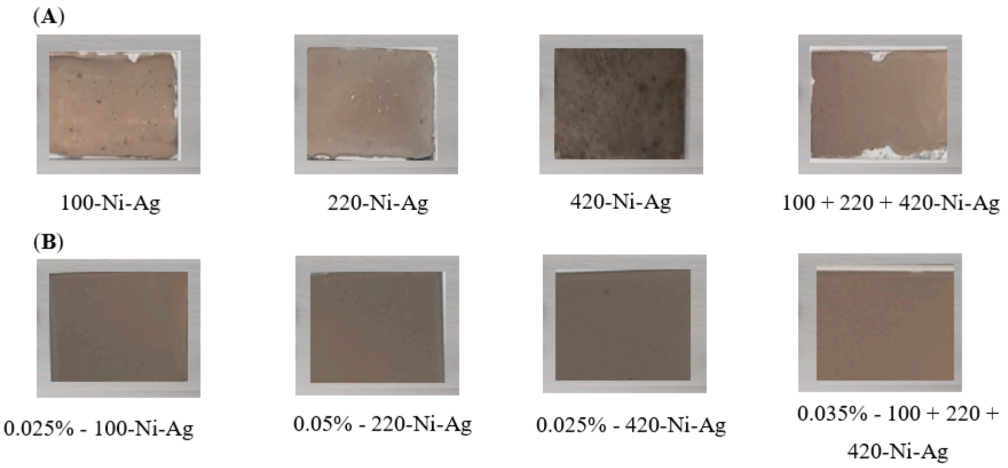

Figure S2. Visualization of the quality of obtained metallic coatings composed of Ni-Ag NPs with different average sizes and the mixture of them, after drying (60 °C, 10 min): (**A**) without wetting agent; (**B**) with the optimal concentration of TEGO WET KL 245 as a wetting agent.

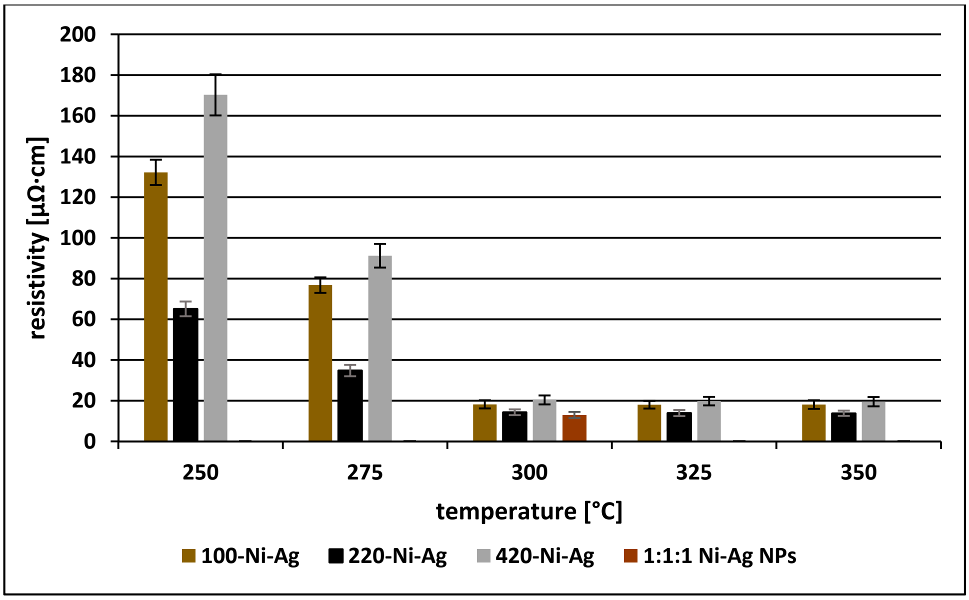

Figure S3. The dependence of the resistivity of metallic films based on Ni-Ag NPs with different average sizes and their 1:1:1 mixture on sintering temperature of (heating duration 30 min).

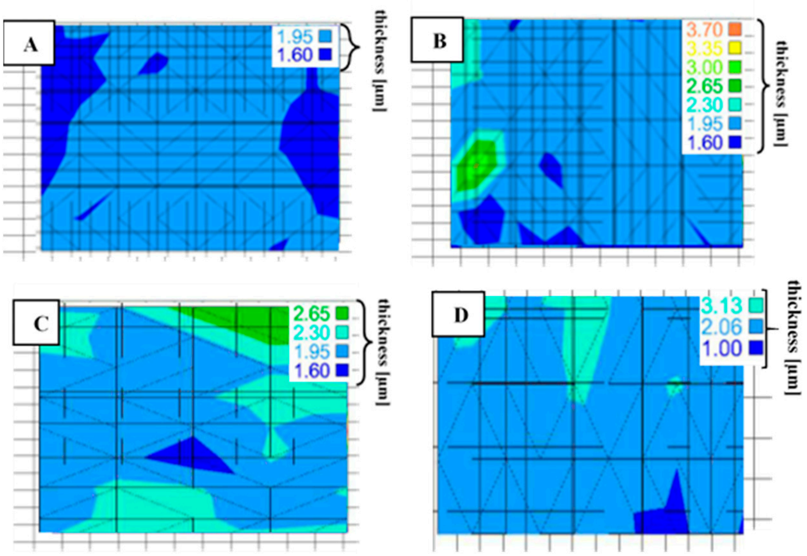

Figure S4. The thickness of the ink coatings composed of Ni-Ag NPs with average sizes (**A**) 100 nm; (**B**) 220 nm; (**C**) 420 nm and (**D**) their mixture after the sintering measured by the EDXRF method.
